# Rapid Low-Cost Production of a Patient-Specific Thumb Orthosis Using Smartphone Light Detection and Ranging (LiDAR) Scanning and Three-Dimensional (3D) Printing

**DOI:** 10.7759/cureus.106955

**Published:** 2026-04-13

**Authors:** Konstantinos Papadakis, Rene D Mileva-Popova, Krasimir K Yanev, Dimitar Peychinov, Todor G Bogdanov

**Affiliations:** 1 Department of Physiology and Pathophysiology, Medical University-Sofia, Sofia, BGR; 2 Department of Dermatology and Venerology, Medical University-Sofia, Sofia, BGR; 3 Department of Surgery, St. Ekaterina University Hospital, Sofia, BGR; 4 Department of Medical Physics, Medical University-Sofia, Sofia, BGR

**Keywords:** 3d printing, additive manufacturing, immobilization, lidar, orthosis, patient-specific, patient-specific thumb orthosis, point-of-care, thumb orthosis

## Abstract

Three-dimensional (3D) printing technologies are increasingly used to produce patient-specific orthoses. Traditional manufacturing methods, such as plaster casting, are fast to apply but produce heavy, non-removable, and poorly ventilated immobilization devices. Many digital workflows for producing custom orthoses require expensive scanners and commercial software, limiting their widespread clinical use. This technical report presents a rapid, low-cost workflow for producing a patient-specific orthosis using a smartphone's light detection and ranging (LiDAR) scanner, free software, and fused deposition modeling (FDM) 3D printing.

The workflow included 3D scanning of the injured limb using an iPhone 15 Pro LiDAR scanner (Apple Inc., Cupertino, CA, USA), mesh processing and region selection in Meshmixer (Autodesk, Inc., San Rafael, CA, USA), orthosis shell generation in Shapr3D (Shapr3D Zrt., Budapest, Hungary) with a shell thickness of 2-2.4 mm, integration of fixation elements for Velcro straps, print preparation, and additive manufacturing using an FDM 3D printer (Bambu Lab X1C; Bambu Lab, Shenzhen, China) with polylactic acid (PLA) filament. The orthosis was designed for thumb immobilization and fixed using three Velcro straps positioned around the wrist, thumb, and metacarpal region.

The total production time from scanning to orthosis placement was approximately three hours. The scanning and digital modeling process took less than one hour, while the 3D printing process was the main time-consuming step. The final orthosis weighed 43 g, was ventilated, removable, and appeared to provide adequate immobilization of the thumb and wrist.

The presented workflow enables rapid, low-cost, and accessible production of patient-specific orthoses using consumer-grade hardware and free software. The method enables same-day orthosis production and may be particularly useful in emergency departments, outpatient clinics, and educational settings where rapid, personalized immobilization is required.

## Introduction

Immobilization orthoses are widely used in the treatment of fractures, ligament and tendon injuries, and postoperative upper-limb stabilization. Traditional immobilization methods include plaster casts and thermoplastic splints, which are effective but have several well-known disadvantages, including relatively high weight, lack of ventilation, hygiene difficulties, and limited possibility for removal and adjustment during treatment. Conventional plaster casts are also associated with skin complications, pressure sores, and patient discomfort due to their closed structure and prolonged immobilization [1].

In recent years, three-dimensional (3D) printing technologies have been increasingly used to produce patient-specific orthoses and casts. Additive manufacturing enables the fabrication of anatomically fitted, lightweight, and ventilated orthotic devices that improve patient comfort and treatment compliance [2,3]. Several studies have demonstrated that 3D-printed orthoses provide comparable mechanical stability to conventional casts while offering advantages such as reduced weight, improved ventilation, and better hygiene [4,5].

The standard digital workflow for producing a patient-specific orthosis typically includes 3D scanning of the affected limb, computer-aided design (CAD) modeling, and additive manufacturing (layer-by-layer fabrication using a 3D printer) [6]. This workflow enables precise anatomical fitting and reproducible orthosis production. However, many published workflows rely on professional 3D scanners, computed tomography, or commercial CAD software, which increases costs and limits the method's accessibility in smaller clinics and emergency settings [2,6].

Recent developments in smartphone technology have introduced light detection and ranging (LiDAR) sensors capable of capturing 3D surface geometry with sufficient accuracy for orthotic applications. LiDAR systems operate by emitting laser pulses and measuring the time-of-flight of reflected signals to reconstruct detailed surface geometry. Recent studies have shown that smartphone-based LiDAR scanners can achieve millimeter-level accuracy, making them suitable for clinical applications such as anatomical modeling, surgical planning, and custom orthosis fabrication. Combined with free mesh processing and CAD software, this enables the development of low-cost and accessible workflows for producing patient-specific orthoses directly at the point of care. Point-of-care manufacturing using 3D printing has the potential to significantly reduce production time and enable same-day fabrication of patient-specific immobilization devices [3].

The novelty of the proposed workflow lies in the use of smartphone-based LiDAR scanning combined with free software and consumer-grade hardware to enable rapid, low-cost, same-day production of patient-specific orthoses at the point of care. This technical report presents a rapid, low-cost digital workflow for producing a patient-specific thumb immobilization orthosis using a smartphone LiDAR scanner, free software, and fused deposition modeling (FDM) 3D printing. It evaluates the method's production time and practical implementation.

## Technical report

Workflow overview

**Figure 1:**
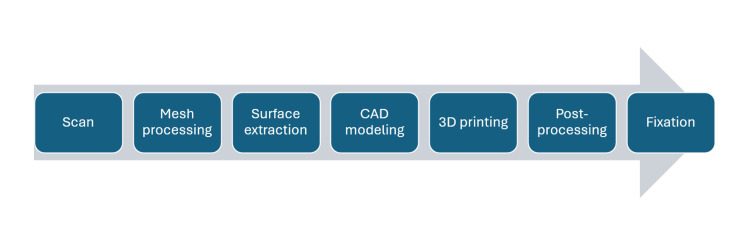
Digital workflow for rapid production of a patient-specific orthosis. CAD: computer-aided design; 3D: three-dimensional

The production of the patient-specific orthosis followed a fully digital workflow consisting of 3D scanning, mesh processing, surface extraction, shell generation, additive manufacturing, and orthosis fixation (Figure 1). The workflow was designed to use accessible consumer hardware and free software to minimize costs and production time. The complete workflow included the following steps: 3D scanning, STL export, mesh processing, CAD modeling, print preparation, 3D printing, post-processing, and orthosis fixation.

3D scanning

**Figure 2:**
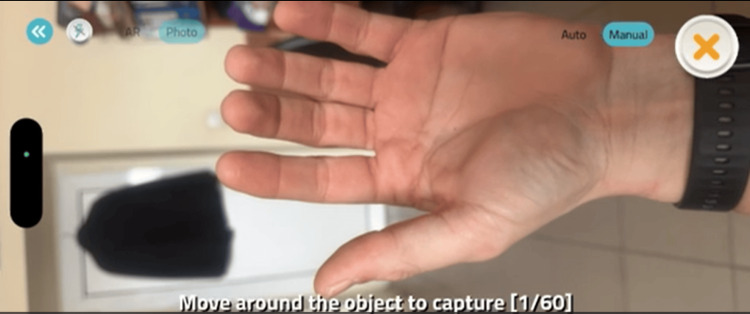
3D scanning of the hand using a smartphone LiDAR sensor. 3D: three-dimensional; LiDAR: light detection and ranging

The injured hand was scanned using an iPhone 15 Pro equipped with a LiDAR sensor (Apple Inc., Cupertino, CA, USA; Figure 2). The scanning was performed by moving the smartphone around the hand in a circular motion to capture the full geometry of the hand, thumb, and wrist. The hand was positioned in a functional immobilization position during scanning to ensure proper orthosis fit. The scanning process took approximately 10-15 minutes. The generated 3D model was exported as an STL file and used for further mesh processing and orthosis design. The anatomical geometry of the hand, thumb, and wrist was captured using a LiDAR-equipped smartphone in a functional immobilization position.

Mesh processing and region selection

**Figure 3:**
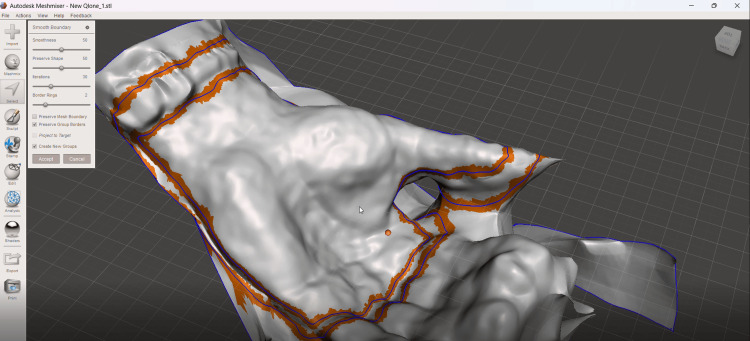
Selection of the immobilization region in the mesh processing software.

The scanned STL model was imported into Meshmixer (Autodesk, Inc., San Rafael, CA, USA) for mesh processing. The region corresponding to the immobilization area was manually selected using the software's selection tools. The selected region included the thumb, the first metacarpal region, and the wrist area, depending on the required immobilization. Тhe region corresponding to the immobilization area was manually selected using the software's selection tools, including the thumb, first metacarpal region, and wrist, which are critical for ensuring proper stabilization and anatomical fit (Figure 3). The region corresponding to the thumb, first metacarpal, and wrist was selected for orthosis design.

After defining the region of interest, the selected surface was extracted and separated from the original mesh. The extracted surface was smoothed to remove scanning artifacts and irregularities that could cause discomfort or pressure points. The resulting surface represented the contact interface between the orthosis and the patient’s skin and was exported as a separate STL file for further CAD modeling.

Orthosis design and shell generation

**Figure 4:**
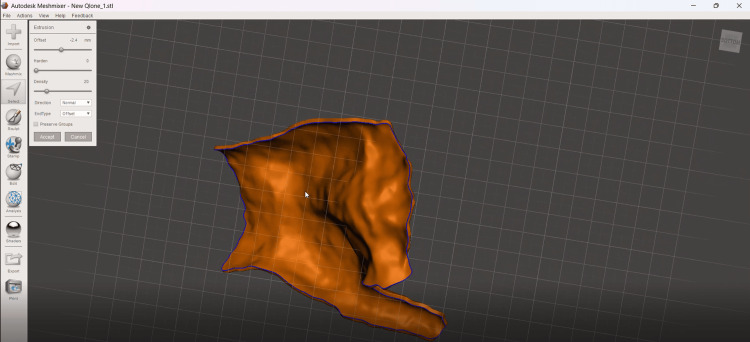
Orthosis shell generated by extrusion of the extracted surface with a thickness of 2–2.4 mm.

The extracted surface was imported into Shapr3D (Shapr3D Zrt., Budapest, Hungary), where the orthosis shell was generated by extruding the surface with a thickness of 2-2.4 mm. This thickness was selected as a compromise between mechanical stability, orthosis weight, and printing time. The orthosis shell was generated by extrusion of the extracted surface with a thickness between 2 and 2.4 mm (Figure 4), providing a balance between mechanical stability, weight reduction, and printing efficiency.

**Figure 5:**
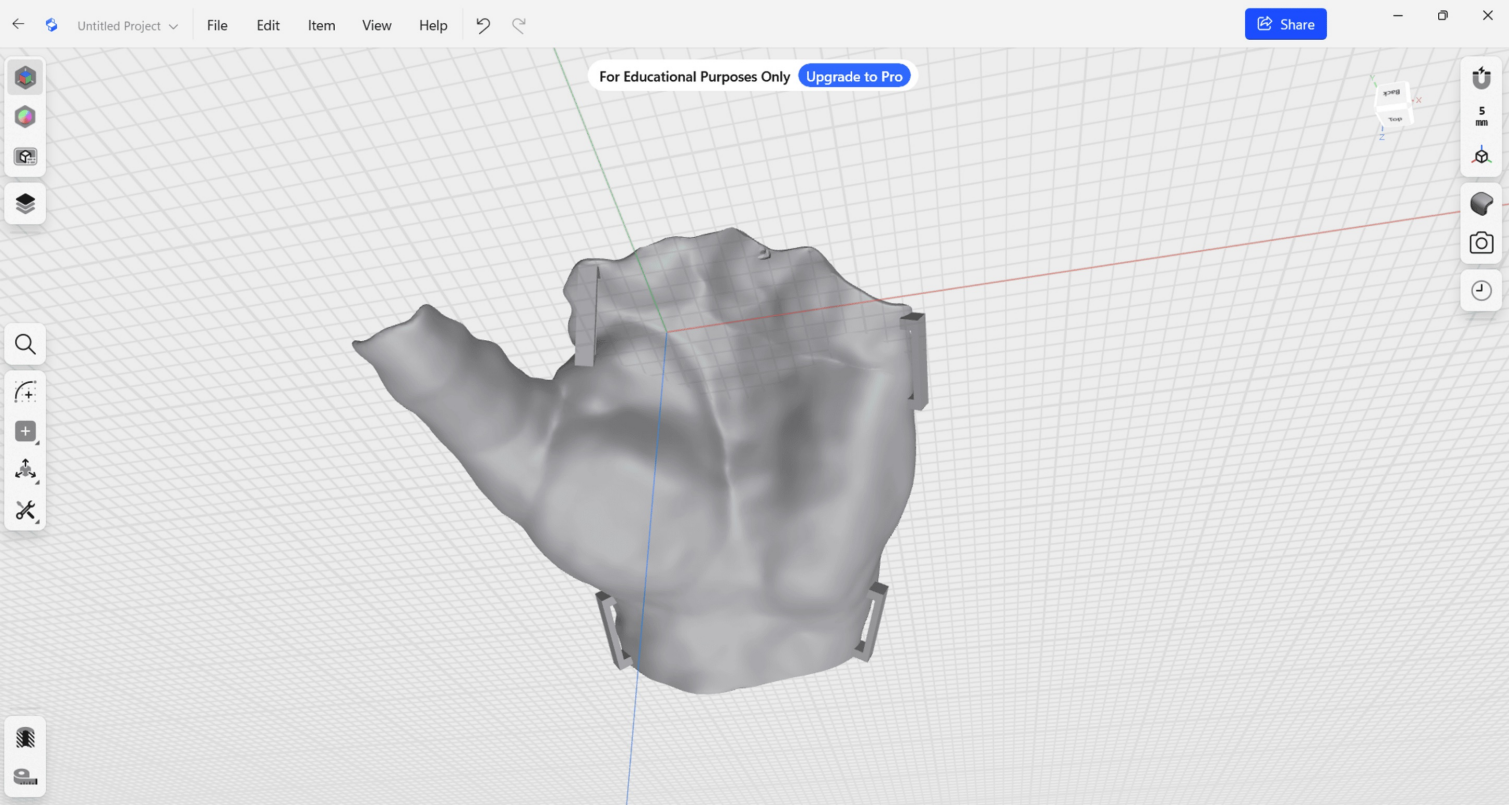
CAD model of the orthosis showing integrated fixation elements for the Velcro straps. CAD: computer-aided design

Additional structural elements were added to the shell model to allow fixation using Velcro straps, ensuring secure attachment and adjustable stabilization of the orthoses. These elements were created by adding predefined geometric features positioned over the shell and merging with the main orthosis structure. Ventilation openings and rounded edges were incorporated into the design to improve comfort, reduce weight, and allow air circulation. The orthosis was designed to be fixed using three Velcro straps positioned around the wrist, the thumb, and the metacarpal region. Additional structural elements were added to allow fixation using Velcro straps (Figure 5).

Print preparation and additive manufacturing

The final STL model was imported into slicing software for print preparation. The model orientation was selected to minimize support structures and reduce printing time while maintaining sufficient mechanical strength. The orthosis was manufactured via FDM with a Bambu X1C 3D printer (Bambu Lab X1C; Bambu Lab, Shenzhen, China) and polylactic acid (PLA) filament. The printing parameters were as follows in Table 1. These parameters were selected to achieve a balance between print speed, structural integrity, and patient comfort.

**Table 1:** 3D printing parameters used for orthosis fabrication. PLA: polylactic acid; 3D: three-dimensional

Parameter	Value
Printer	Bambu X1C (Bambu Lab X1C; Bambu Lab, Shenzhen, China)
Nozzle diameter	0.4 mm
Layer height	0.2 mm
Infill	20%
Material	PLA
Printing time	~2 hours
Orthosis weight	43 g

**Figure 6:**
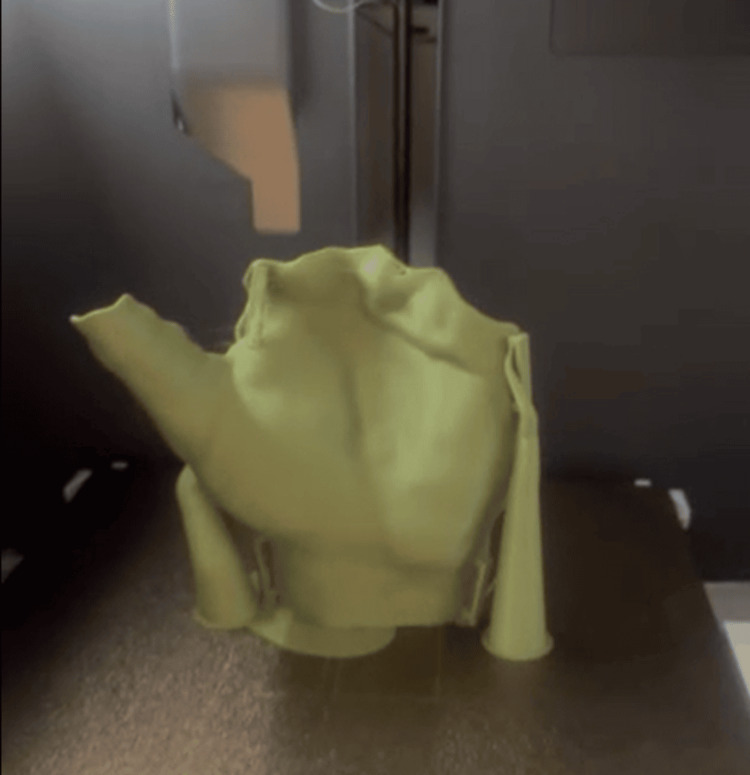
3D printed orthosis manufactured using FDM printing with PLA filament. 3D: three-dimensional; FDM: fused deposition modeling; PLA: polylactic acid

PLA was selected due to its ease of printing, low cost, and sufficient rigidity for temporary immobilization. Other materials, such as polyethylene terephthalate glycol (PETG) or acrylonitrile butadiene styrene (ABS), may be used depending on the required mechanical properties and clinical application. The orthosis was manufactured using FDM 3D printing with PLA filament (Figure 6).

Post-processing and fixation

**Figure 7:**
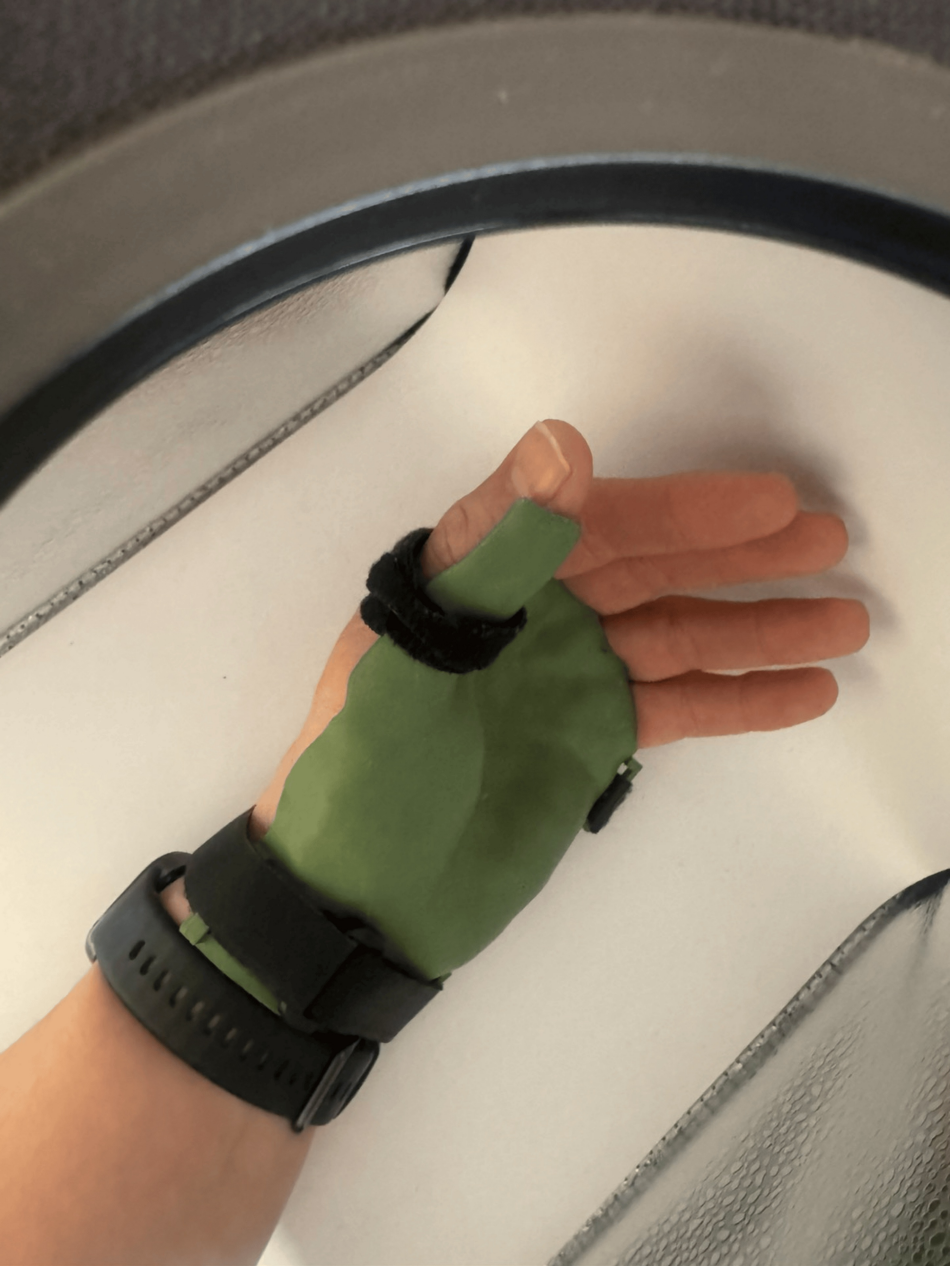
Final orthosis positioned on the patient’s hand and fixed using three Velcro straps.

After printing, the support structures were removed manually. The edges were smoothed where necessary to improve patient comfort. Velcro straps were inserted through the integrated guiding elements of the orthosis to form the fixation system. The orthosis was fixed using three Velcro straps around the wrist, around the thumb, and above the fingers. This fixation system allowed stable immobilization of the thumb and wrist while allowing partial movement of the other fingers. The orthosis could be removed and reattached, allowing hygiene maintenance and skin inspection. The orthosis was fixed using three Velcro straps positioned around the wrist, thumb, and metacarpal region (Figure 7).

Production time

The total production time from scanning to orthosis placement was approximately three hours. Production time frame is presented in Table 2.

**Table 2:** Time distribution for each stage of orthosis production. 3D: three-dimensional; CAD: computer-aided design; min: minutes; h: hours

Step	Time
3D scanning	10–15 min
Mesh processing	15–20 min
CAD modeling	20–25 min
Print preparation	10 min
3D printing	~2 h
Post-processing and fitting	10–15 min

**Figure 8:**
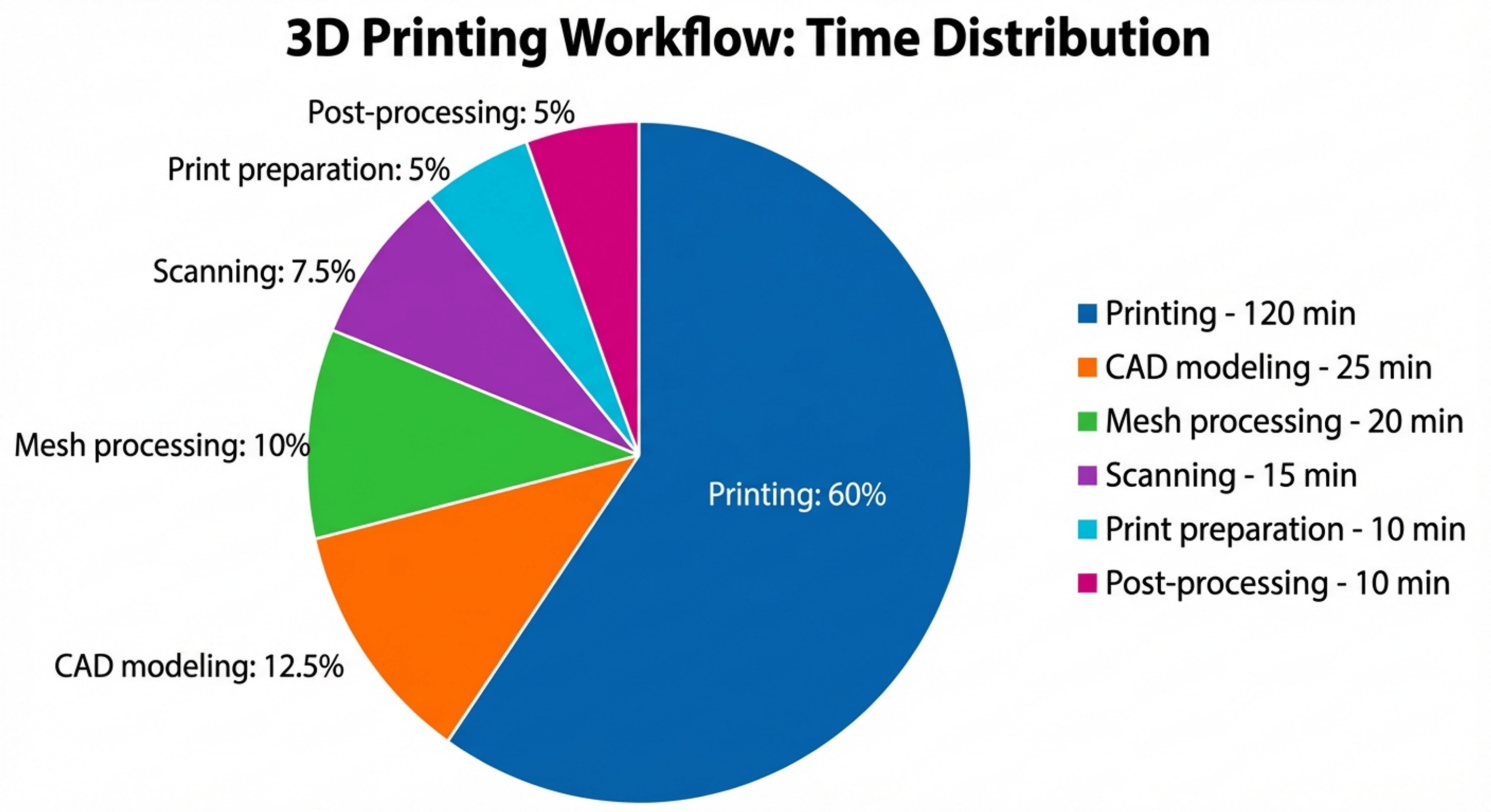
Distribution of production time across the different stages of the orthosis manufacturing process. 3D: three-dimensional; CAD: computer-aided design; min: minutes

The results highlight that the 3D printing stage is the most time-consuming step, while digital preparation remains relatively short. The digital preparation time was less than one hour and remained relatively constant regardless of the orthosis geometry, whereas the 3D printing process was the main time-limiting step. The distribution of production time across the different stages of orthosis manufacturing is shown in Figure 8. The 3D printing process was the most time-consuming step, while scanning and digital modeling required less than one hour.

Cost analysis

The orthosis weighed 43 g and was manufactured using PLA filament. The material cost per orthosis was approximately 1 to 2 Euros. The software used in the workflow was free, and the hardware consisted of consumer-grade devices (a smartphone and a desktop 3D printer), which significantly reduced the overall cost of production compared to traditional CAD/computer-aided manufacturing (CAM) orthosis manufacturing systems.

## Discussion

The present technical report describes a rapid and low-cost workflow for producing a patient-specific thumb immobilization orthosis using smartphone LiDAR scanning, free software, and FDM 3D printing. The main contribution of this work is not the orthosis itself but the simplified, accessible digital workflow that enables same-day orthosis production with consumer-grade hardware and free software.

Traditional immobilization methods, such as plaster casts and thermoplastic splints, are widely used due to their fast application and low material cost, but they have several well-known disadvantages. Plaster casts are relatively heavy, non-removable, and poorly ventilated, which may lead to skin irritation, pressure sores, hygiene difficulties, and patient discomfort during long-term immobilization [7]. In addition, the traditional fabrication process depends heavily on the clinician’s experience and is difficult to reproduce once the cast is removed [8].

3D printing technologies enable the production of patient-specific orthoses that are lightweight, ventilated, removable, and anatomically fitted. Previous studies have reported that 3D-printed orthoses can provide adequate immobilization while improving patient comfort and satisfaction compared to conventional casts [9-11]. For example, a clinical study on 3D-printed casts for distal radius fractures reported high patient satisfaction, effective fracture immobilization, and the absence of pressure-related complications, with patients preferring the 3D-printed cast to the traditional plaster cast [9]. Similarly, randomized studies comparing 3D-printed splints with thermoplastic splints have demonstrated comparable immobilization performance and high patient satisfaction [10,11]. A comparison between conventional plaster casts and 3D-printed orthoses is presented in Table 3, highlighting key advantages such as reduced weight, improved ventilation, and better hygiene.

**Table 3:** Comparison between conventional plaster cast and 3D printed orthosis. 3D: three-dimensional

Parameter	Plaster cast	3D printed orthosis
Weight	High	Low (43 g)
Removable	No	Yes
Ventilation	No	Yes
Hygiene	Poor	Good
Skin inspection	No	Yes
Reproducibility	No	Yes
Production time	Short application time	~3 hours
Customization	Limited	Patient-specific

Another important advantage of 3D printing in orthosis production is the ability to achieve patient-specific customization and improved anatomical fit. Customized orthoses have been shown to improve comfort, function, and patient compliance due to better anatomical matching and reduced weight [3]. In addition, the ventilated structure of 3D-printed orthoses reduces the risk of skin complications and improves hygiene during immobilization [12]. These characteristics are particularly important for upper limb immobilization, where hygiene and skin monitoring are essential.

The standard digital workflow for producing a patient-specific orthosis includes 3D scanning, CAD modeling, and additive manufacturing [8]. However, many published workflows rely on professional 3D scanners, computed tomography, or commercial CAD software, which increase both cost and production time and limit the accessibility of the method [4,8]. In contrast, the workflow presented in this report uses a smartphone LiDAR scanner and free software, which significantly reduces the cost and simplifies the digital modeling process while maintaining sufficient accuracy for orthosis fabrication.

Production time is one of the main limitations reported in the literature for 3D-printed orthoses. Many previously published workflows require multiple clinical visits and waiting times ranging from several days to several weeks for the fabrication of custom orthoses [4]. In the present report, the total production time was approximately three hours, with less than one hour for scanning and digital modeling and approximately two hours for 3D printing. This demonstrates that patient-specific orthoses can be produced on the same day, which represents a significant advantage in emergency and outpatient settings. From a practical perspective, this production time enables same-day orthosis fabrication, which may significantly improve clinical workflow efficiency compared to conventional methods that often require multiple visits and longer waiting times.

Another important aspect of this workflow is the use of point-of-care manufacturing. Point-of-care 3D printing enables medical devices to be designed and manufactured directly at the point of care, reducing production time and improving clinical workflow efficiency [13]. With the decreasing cost of 3D printers and the availability of free software, point-of-care manufacturing is expected to become increasingly common in clinical practice. It may significantly change the way immobilization devices are produced and delivered to patients.

The orthosis presented in this report weighed 43 g, which is significantly lower than the weight of conventional plaster casts reported in the literature. This reduced weight may contribute to improved patient comfort and compliance during immobilization. Previous studies have shown that 3D-printed casts may weigh up to ten times less than traditional casts while maintaining sufficient mechanical stability [12]. Reduced weight is associated with improved patient comfort and better treatment compliance. A comparison of the presented workflow with published studies on 3D-printed orthoses can be seen in Table 4.

**Table 4:** Comparison of the presented workflow with published studies on 3D printed orthoses. 3D: three-dimensional; CT: computed tomography; CAD: computer-aided design; LiDAR: light detection and ranging

Study	Scanner	Software	Production time	Weight
Li et al. [8]	3D scanner	CAD	Several hours	Not reported
Chen et al. [9]	CT	CAD	1–2 days	200–300 g
Lin et al. [12]	3D scanner	CAD	Several hours	~150 g
Present study	Smartphone LiDAR	Free software	~3 hours	43 g

The main limitation of the presented workflow is the 3D printing time, which remains the longest step in the process. In addition, PLA material may not be suitable for long-term immobilization or high-load applications, and other materials such as PETG, ABS, or nylon may be more appropriate in certain clinical situations. Another limitation is the accuracy of smartphone LiDAR scanning, which may be lower than that of professional medical scanners; however, the Velcro-strap fixation system allows small geometric deviations to be compensated during fitting.

In addition, the present work is primarily focused on demonstrating technical feasibility and accessibility rather than providing quantitative validation. No mechanical testing, geometric accuracy analysis, or clinical outcome evaluation was performed. Future studies should include systematic validation of mechanical performance, reproducibility, and clinical effectiveness, as well as patient-reported outcomes such as comfort, usability, and satisfaction.

Overall, the results of this report suggest that patient-specific orthoses can be produced rapidly and at low cost using accessible consumer technology. The presented workflow demonstrates that same-day production of lightweight, removable, and ventilated orthoses is feasible and may represent a practical alternative to traditional immobilization methods in selected clinical cases.

## Conclusions

This technical report presents a rapid and low-cost digital workflow for producing a patient-specific thumb immobilization orthosis using smartphone LiDAR scanning, free software, and FDM 3D printing. The presented method enables same-day orthosis production, with a total production time of approximately three hours and less than one hour required for scanning and digital modeling. The produced orthosis was lightweight, ventilated, and removable, providing functional immobilization with a three-strap fixation system. The workflow uses accessible consumer hardware and free software, significantly reducing production costs and supporting point-of-care implementation.

The main advantage of the presented approach is the combination of rapid production time, low cost, and reproducibility of the digital workflow. Compared to traditional plaster immobilization and conventional custom orthosis fabrication methods, the workflow demonstrates potential benefits such as faster production, improved hygiene, reduced weight, and easy modification of the orthosis design. While these findings highlight the feasibility and practical potential of the approach, further studies are required to evaluate its clinical effectiveness, safety, and generalizability. This method may have potential applications in emergency departments, outpatient clinics, and educational settings, and it demonstrates the promise of point-of-care additive manufacturing for patient-specific immobilization devices.
